# Move and Play Programme: Perceptions of Paediatric Physiotherapists and Parents of Infants

**DOI:** 10.1111/cch.70324

**Published:** 2026-08-05

**Authors:** Natália Guimarães Melo, Nayara Rodrigues Gomes de Oliveira, Vanessa Cordeiro de Sousa, Cibelle Kayenne Martins Roberto Formiga

**Affiliations:** ^1^ Department of Physiotherapy, Program in Sciences Applied to Health Products (PPGCAPS) Universidade Estadual de Goiás Goiânia Goiás Brazil

**Keywords:** audiovisual aids, child development, educational technology

## Abstract

**Introduction:**

Educational videos can improve parent engagement in early childhood development. For widespread adoption, these videos need to be practical and of high quality. This study evaluated how paediatric physiotherapists and parents perceived the educational videos of the ‘Move and Play Programme’.

**Methods:**

This cross‐sectional study included 209 paediatric physiotherapists and 100 parents, with data collected online. Each participant received links to two or three videos and an evaluation form. Videos were rated for presentation, content and objectives. Physiotherapists assessed clinical applicability, whereas parents rated home use. Descriptive statistics were used to analyse profiles and perceived video quality.

**Results:**

Most videos were rated as fully adequate in terms of presentation, content and clarity of objectives by both groups. Physiotherapists found the videos to be useful for guiding home‐based activities and communicating with families. Parents reported that the videos supported their infants' development and increased their confidence in performing activities independently. Most would recommend the videos.

**Conclusion:**

The ‘Move and Play Programme’ videos are effective tools for home developmental activities, recommended by both parents and paediatric physiotherapists.

## Summary

- Accessible educational tool with potential for practical application: The Move and Play Programme videos were rated as clear, simple and easy to apply by paediatric physiotherapists and parents, confirming their potential as an educational resource for promoting early child development.
- Integration between clinical and home environments: The videos strengthen the connection between professional guidance and family practice, allowing activities to be performed at home using simple, easily accessible materials by parents and caregivers.
- High acceptability and cross‐cultural applicability: The programme demonstrates strong acceptability and has the potential to be implemented in diverse health contexts and by professionals from other countries through its publicly accessible platform.
- Potential for incorporation into public health policies and telehealth services: Given its accessibility and positive reception, the Move and Play Programme can be integrated into primary care and telehealth initiatives, expanding the reach of preventive and educational strategies aimed at families of infants at risk for developmental delays.
- Future perspectives for equity and impact: Future research should include families from diverse socioeconomic and cultural backgrounds, assessing the long‐term effects on child development and professional practice, thereby promoting greater equity in access to health information.

## 1 Introduction

Child development is a dynamic and continuous process characterized by the progressive acquisition of motor, cognitive, socioemotional and communicative skills that enable the child to interact with the environment and adapt to the demands of the context in which they live (Hadders‐Algra 2018; Zubler et al. 2022). However, this process may be negatively influenced by biological and environmental factors such as prematurity, low birth weight, neonatal complications and unfavourable socioeconomic conditions, which are associated with long‐term developmental impairments (Fernandes et al. 2022; Formiga et al. 2017; Fuentefria et al. 2017; Silva et al. 2026). Given these risk factors, early interventions and developmental promotion strategies become essential, especially during the first year of life.

In this context, parental participation is recognized as a central element in promoting child development. The literature demonstrates that parent‐guided interventions can favour positive motor and cognitive outcomes, particularly in populations with greater socioeconomic vulnerability (Luoto et al. 2021; Muhoozi et al. 2018). However, parental knowledge regarding child development is not always sufficient to sustain appropriate practices in the home environment, especially when the guidance provided by professionals is not fully understood or easily applicable to the family routine (Lee et al. 2020). Therefore, beyond providing guidance, it is necessary to offer educational materials that help parents translate technical knowledge into practical actions in the child's daily life (Khurana et al. 2020; Pedersen and Hansen 2022; Trubian et al. 2022).

Educational technologies have emerged as promising tools in maternal and child health, as they expand access to information, strengthen communication between families and health services and promote adherence to professional guidance (Higgs et al. 2014). Educational materials that include visual resources, such as illustrations, images and videos, facilitate the association between theoretical and practical information, contributing to better understanding and retention of content. Previous studies have demonstrated positive results using different formats of educational materials, such as comic books (Rosas‐Blum et al. 2018) and educational videos developed by health professionals (Corrêa et al. 2024).

At the same time, the increasing use of the internet and mobile devices has transformed how parents seek health information. Digital resources such as applications and online videos have become frequent sources of consultation due to their practicality and compatibility with family routines (Meyers et al. 2020; Pluye et al. 2025; Zhang et al. 2020). However, the wide availability of online content raises concerns regarding the quality and reliability of information, especially in paediatrics, where studies have identified the presence of materials with low scientific validity (Bakilan et al. 2023; Furtado et al. 2022; Cavalcante et al. 2024).

Although initiatives for the development of educational technologies in health exist, there remains a gap in the literature regarding the production and systematic evaluation of educational videos specifically aimed at promoting child development during the first year of life, developed based on scientific evidence and evaluated by both specialists and users. In light of this need, the present study aims to evaluate the perception of paediatric physiotherapists and parents regarding the quality of the ‘Move and Play Programme’, composed of educational videos designed to support infant development during the first year of life.

## 2 Methods

This is a cross‐sectional observational study aimed at evaluating the quality of the Move and Play Programme, composed of educational videos designed to support infant development during the first year of life. This study was conducted in accordance with the Guidelines and Regulatory Standards for Research Involving Human Subjects and was approved by the Human Research Ethics Committee.

The ‘Move and Play Programme’ was developed by Brazilian researchers and consists of 59 educational videos narrated in Brazilian Portuguese and subdivided accordingly. The videos were organized into four age groups (0–3 months; 4–6 months; 7–9 months; and 10–12 months) and distributed across five activity categories: personal, social and communication; gross motor; fine‐adaptive motor; sensory aspects; and the production of homemade materials or resources.

For the development of the educational videos, widely recognized instruments for assessing development in this age group were used as references: the Alberta Infant Motor Scale (AIMS), the Denver Developmental Screening Test II (Denver II) and The Survey of Well‐being of Young Children, Brazilian version (SWYC‐BR). These instruments supported the selection of the main developmental milestones expected for each age group.

After this stage, the videos were recorded with model infants, following previously structured scripts based on these milestones. Subsequently, the images were edited by selecting the best scenes and adding narration using simple, accessible language in Brazilian Portuguese. Initially, the videos were evaluated by specialists, and the final version was later analysed by parents and physiotherapists in the present study.

The titles of the videos that compose the programme, organized according to the five proposed categories, are presented in Table 1. Each video has an average duration of approximately 1 min and 44 s and presents activities and skills related to child development during the first year of life, which can be performed in the home environment according to the expected milestones for each age group.

**TABLE 1:** Activities and skills that make up the ‘Move and Play Programme’, subdivided into categories and showing the title of each video created by the authors (*n* = 59 videos).

Skill characteristics	Video title
Personal, social, communication	How to carry your baby
	Observing and imitating sounds and facial expressions
	Playing on the lap
	Interacting with your baby
	Sleep positions
	Playing while changing clothes
	Bath time positions
	Playing in the bathtub
	Playing ball
	Playing clapping and waving bye‐bye
	Playing repeating words
	Playing peek‐a‐boo
	Starting to eat independently
	Learning to use a cup
	Playing to imitate animals
Gross motor	Stimulating head control
	Tummy time
	Playing in the lying position
	Playing the bicycle kicks
	Playing with hands to feet
	Playing to rolling
	Playing with hands
	Playing *serra‐serra*
	Pulling to sit
	Practising sitting
	Playing in the sitting position
	Different ways of sitting
	Playing to pivot
	Learning to transition from sitting to hands‐and‐knees position
	Playing in the hands‐and‐knees position
	Playing to crawl
	Playing in the kneeling position
	Playing in the half‐kneeling position
	Learning to stand
	Playing in the standing position
	Playing to stand alone
	Playing to walk sideways
	Starting to walk forward
Fine‐motor adaptive	Playing to track objects with the head
	Playing to reach objects
	Playing with two objects
	Playing with small objects
	Playing with clothespins
	Playing with a pasta strainer
	Playing to tower cubes
	Activities with adhesive tape
	Playing with an egg carton
	Playing to pull velcro
Sensorial aspects	Playing to recognize body parts
	Playing with textures
	Learning hand‐knee, hand‐foot and foot‐foot coordination
	Learning hand‐hand coordination
	Playing in front of the mirror
Material production	Learning to make a toy with an egg carton
	Learning to make a toy with velcro
	Learning to make a rattle at home
	Producing and using the pants of grandma
	Learning to make towel rolls
	Learning to make visual stimulation cards

*Source:* Authors (2025).

Brazilian paediatric physiotherapists and parents of infants under 12 months evaluated the ‘Move and Play Programme’. Licensed physiotherapists working in child health and parents from across Brazil were recruited for this study. Participants first consented digitally. Only those who submitted evaluations on time were included.

All stages of research were completed remotely. Researchers began by sharing an invitation via social media, which included posts designed to recruit both parents and physiotherapists. Interested candidates completed a Google Forms questionnaire, providing basic details such as name, contact information and participant category. Completion signalled their interest in participating.

After confirming interest through the initial form, researchers contacted each participant with a second link containing detailed study information. This second step provided access to the digital Informed Consent Form (ICF), an ID survey, a set of two or three randomly assigned videos and a video review form. Participants evaluated only those videos assigned to them.

Researchers selected videos at random in advance using Excel. This made sure all programme videos were reviewed. Each video was watched at least 10 times and up to 14 times by physiotherapists and at least 4 and up to 6 times by parents. When the videos were distributed to parents, the specific age of the child was not taken into account. The only criterion was that the participant had at least one child under 1 year of age, and the videos were assigned based on prior randomization.

**FIGURE 1:**
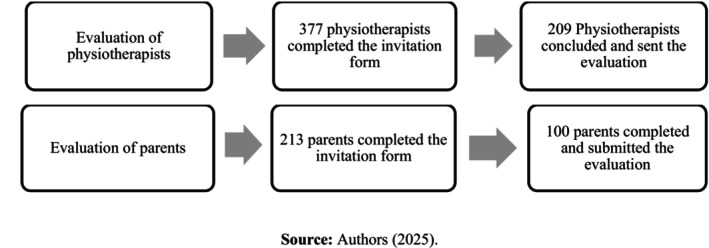
Organizational chart showing the sampling route during the video evaluation stage by physiotherapists and parents/caregivers.

Figure 1 outlines the flow of participants through the study, from their receipt of the social media invitation, expression of interest, completion of consent, assignment of videos, to submission of video reviews. This clarifies each step of participant involvement.

The following instruments were used to meet the objectives of this study:

- Identification questionnaire—physiotherapists and parents/caregivers: Developed by the researchers and administered online, this questionnaire included both open‐ and closed‐ended questions to collect participant information. For physiotherapists, 13 questions were designed to gather data such as name, age, sex, marital status, education level, years of experience in paediatric care and city and state of practice. For parents, 20 questions were developed to collect information including name, age, sex, marital status, education level, place of residence and number of children.
- Video review questionnaire—physiotherapists: Developed by the researchers and administered online, this questionnaire included both open‐ and closed‐ended questions to evaluate the videos (Supporting Information S1). In total, 19 questions were related to the educational videos: 5 addressing presentation, 5 content, 2 objectives, 6 applicability in professional practice and 1 open‐ended question for comments or suggestions. The 18 closed‐ended questions were structured in three formats: (1) yes or no; (2) a 4‐point Likert scale (totally adequate, adequate, partially adequate and inadequate); and (3) a rating scale from 0 (worst) to 10 (best). The questionnaire was developed based on domains and evaluation criteria previously used in studies with similar methodological characteristics (de Campos et al. 2021).
- Video review questionnaire—parents/caregivers: Developed by the researchers and administered online, this questionnaire included both open‐ and closed‐ended questions to evaluate the quality of the videos (Supporting Information S2). In total, 21 questions addressed the educational videos: 5 related to presentation, 5 to content, 2 to objectives, 8 to applicability at home and 1 open‐ended question for comments or suggestions. The structure was the same as that used for physiotherapists: one open‐ended question and three types of closed‐ended questions. The questionnaire was developed based on domains and evaluation criteria previously used in studies with similar methodological characteristics (de Campos et al. 2021).

Data from the identification questionnaire and the evaluation of the videos by parents and physiotherapists were extracted from Google Forms and organized in an Excel spreadsheet according to the nature of the data, which were classified as either nominal or numerical.

After this organization process, the data were analysed using the Statistical Package for the Social Sciences (SPSS), Version 23.0. Descriptive statistics were performed, with means and standard deviations calculated for numerical variables and absolute and relative frequencies calculated for categorical variables.

## 3 Results

The video evaluations by physiotherapists and parents assessed whether the material was accessible, objective, simple and useful. A total of 209 physiotherapists working in child health and 100 parents of infants in their first year participated. Participant characteristics are shown in Table 2. Characterization of physiotherapists working in the area of child health and parents of children in the first year of life is shown in Table 2.

**TABLE 2:** Characterization of physiotherapists working in the area of child health (*n* = 209) and parents of children in the first year of life (*n* = 100).

Characteristics	Physiotherapists (*n* = 209)		Parents (*n* = 100)	
	*f*	%	*f*	%
Sex				
Female	195	93.3	96	96.0
Male	14	6.7	4	4.0
Civil status				
Married	113	54.1	73	73.0
Single	84	40.2	27	27.0
Divorced	11	5.3	—	—
Widowed	1	0.5	—	—
Education				
Doctoral degree (completed)	26	12.4	—	—
Doctoral degree (in progress)	12	5.7	—	—
Master's degree (completed)	30	14.4	—	—
Master's degree (in progress)	18	8.6	—	—
Postgraduate diploma/certificate (completed)	99	47.4	42	42.0
Postgraduate diploma/certificate (in progress)	16	7.7	—	—
Bachelor's degree (completed)	8	3.8	21	21.0
Bachelor's degree (in progress)	—	—	13	13.0
Secondary education (completed)	—	—	15	15.0
Secondary education (incomplete)	—	—	4	4.0
Primary education (completed)	—	—	1	1.0
Primary education (incomplete)	—	—	4	4.0
Regions of Brazil				
North	11	5.3	—	—
Northeast	27	12.9	2	2.0
Central‐West	73	34.9	89	89.0
Southeast	71	34.0	9	9.0
South	27	12.9	—	—
	Mean	SD	Mean	SD
Age (years)	35.6	8.8	29.8	7.6
Length of experience in paediatrics (years)	9.9	8.5	—	—
Number of children	—	—	1.4	0.6

*Note:* Data expressed as frequency (*f*), percentage (%), mean and standard deviation (SD). For some items, the symbol ‘—’ was used, meaning that there was no response for that item/category because it was not applicable or because the result in that category was equal to 0.

*Source:* Author's own (2025).

Most physiotherapists were female (93.3%) and had at least a completed postgraduate qualification (47.4%). They had an average of approximately 10 years of experience in child health. The majority were located in the Central‐West (34.9%) and Southeast (34.0%) regions of Brazil.

The mean age of parents was 29.8 years (±7.6), with most being female (96%). Most had only one child (mean: 1.0 ± 0.6), held a Bachelor's degree (21%) or a Postgraduate Certificate/Diploma (42%), and resided in the Central‐West region of Brazil (89%).

**FIGURE 2:**
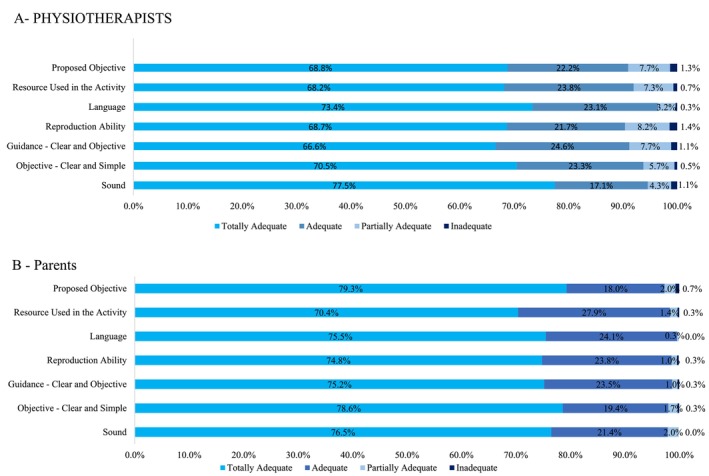
Graphical representation of the evaluation of the videos by physiotherapists and parents, considering the presentation, content and objective of the videos. *Note:* Evaluation by physiotherapists (A) and parents (B) regarding the topics presentation (sound), content (objective, orientation, reproducibility, language and resources used) and objective (proposed objective) of the video. A total of 209 physiotherapists and 100 parents participated in the study, with each parent or physiotherapist evaluating an average of two or three videos randomly, totaling 294 evaluations by parents and 613 evaluations by physiotherapists. Data are expressed as a percentage (%) according to the total number of evaluations in each group (parents or physiotherapists).

Figure 2 presents the results of the video quality assessment. Of the 209 physiotherapists, 195 evaluated three videos and 14 evaluated two, totalling 613 assessments. Among parents, 94 evaluated three videos, and 6 evaluated two, resulting in a total of 294 assessments.

Both physiotherapists and parents predominantly rated the videos as fully appropriate in presentation, content and clarity of objectives. For video duration, rated on a 0–10 scale, the mean score was 9.6 (SD = 0.9), indicating high acceptance.

**FIGURE 3:**
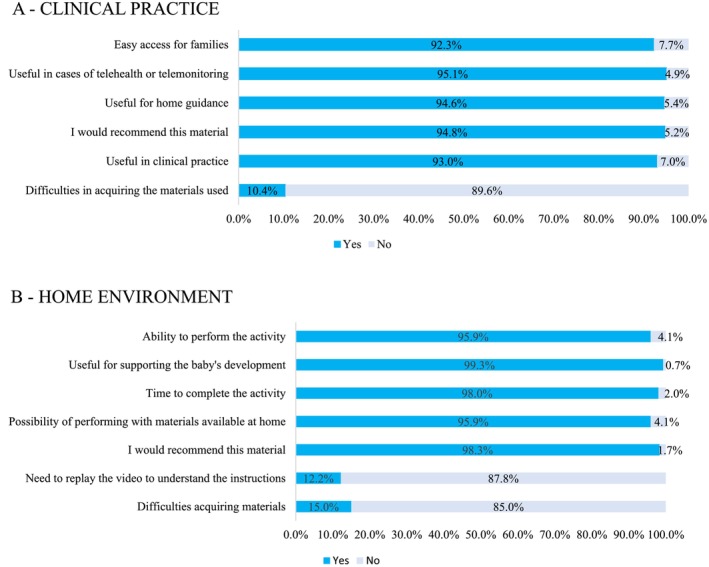
Graphical representation of the results of the video evaluation, regarding applicability in clinical practice (physiotherapists) and applicability in the home environment (parents). *Note:* Assessment by physical therapists (A) and parents (B), regarding applicability in clinical practice and in the home environment. A total of 209 physical therapists and 100 parents participated in the study, with each parent or physical therapist randomly evaluating an average of two or three videos, totalling 294 parental assessments and 613 physical therapist assessments. Data are expressed as a percentage (%) according to the total number of assessments in each group (parents or physical therapists).

Applicability was evaluated from two perspectives. Physiotherapists rated the videos' relevance to clinical practice. Parents rated the feasibility of performing the activities at home. Figure 3 presents these results.

Most videos were considered useful for both clinical practice and at‐home guidance and accessible to families. Physiotherapists rated family understanding on a 0–10 scale, with a mean score of 9.3 (SD = 1.3).

Most parents found the videos to be useful in supporting infant development, accessible to families and feasible to replicate using available toys or easily acquired materials. They also reported that instructions were clear after one viewing, with a mean sufficiency rating of 9.6 (SD = 0.9).

## 4 Discussion

In our study, physiotherapists and parents received the videos to assess presentation, content, objectives and applicability. Physiotherapists contributed technical expertise, whereas parents represented the target audience.

Scientific literature recommends that educational videos be developed in three stages: preproduction (script creation and validation), production (recording and editing) and postproduction (audience evaluation), to ensure methodological rigour and reliability (Barbosa et al. 2023). This study focused on the postproduction stage, evaluating the material from the perspectives of experienced physiotherapists and parents of infants in their first year of life, the primary target audience.

This type of evaluation helps ensure both content clarity and technical accuracy. For example, Santos et al. (2020) evaluated an educational booklet for use in intensive care units with healthcare specialists and mothers, assessing objectives, structure, presentation and relevance. Experts considered the material appropriate, and mothers found it easy to understand. Similarly, in our study, both physiotherapists and parents rated the material as highly appropriate, supporting its potential as a reliable and accessible source of information.

Educational videos have become important tools for disseminating evidence‐based health information. A recent study demonstrated that cancer education videos are effective when tailored to the target audience, considering socioeconomic and cultural factors to ensure broad accessibility (Blee et al. 2022). In our study, parents generally provided positive evaluations of the programme's videos, particularly regarding presentation, content and language. Participants from different regions of Brazil and with varying educational levels were included, indicating high acceptability and accessibility of the material.

Diversity of social contexts is essential in health education, especially for families in vulnerable situations. Similar to our findings, a study by Musiimenta et al. (2021) evaluated a mobile application featuring educational videos and reminders to support maternal and child health among mothers with low educational levels in hard‐to‐reach areas. The videos were considered informative and promoted paternal engagement. In our study, parents also represented diverse backgrounds, with 24% having completed only primary or secondary education. The results suggest that the content was appropriate and accessible across different social contexts.

Although not directly assessed in this study, previous research suggests that watching educational videos about infant development may empower caregivers by improving understanding and encouraging adherence to recommended practices (Schneider et al. 2021). In this context, the ‘Move and Play’ programme has the potential to contribute to caregiver empowerment and health education during the first year of life, which can be further evaluated in future studies.

Regarding limitations, although the study used broad dissemination through social media, parental participation was concentrated in certain regions of Brazil, particularly the Central‐West. This regional concentration may limit the generalizability of the findings, as other regions were underrepresented. Additionally, a relatively high proportion of participants had higher education levels, which may have influenced the ease of comprehension and acceptance of the material. Furthermore, participant dropout was observed, and although the reasons were not formally assessed, this factor should be considered when interpreting the acceptability of the intervention. It is possible that the online format of the study contributed to this dropout, as participation required time to watch the videos and complete the questionnaires. In addition, some parents may have been less engaged due to not knowing the professionals responsible for the study, which may also have acted as a limiting factor. Future studies should aim to include more socioeconomically and geographically diverse samples and investigate the programme's impact among families in more vulnerable contexts.

A key strength of this study is the inclusion of experienced physiotherapists, which contributes to the technical validity of the evaluation. The participation of parents, as the primary target audience, adds practical relevance regarding usability and comprehension in home settings. In addition, the organization of videos according to age and developmental skills, combined with a structured evaluation process, strengthens the quality of the ‘Move and Play’ programme as an educational resource.

The findings suggest that the ‘Move and Play’ programme can support both healthcare professionals and families in promoting infant development during the first year of life. The videos are accessible, use clear language and facilitate the integration of developmental activities into the home routine, encouraging parents to take an active role. Moreover, healthcare professionals may use these videos as supportive tools in telehealth or remote care contexts.

The programme also demonstrates strong potential for integration into public health policies and primary care, contributing to preventive and educational strategies in early childhood development. Future research should focus on more diverse populations and assess long‐term outcomes related to child development and professional practice. Demonstrating sustained benefits will further support the relevance and adaptability of the ‘Move and Play’ programme across different contexts.

## 5 Conclusion

The findings show that the ‘Move and Play’ programme videos were enthusiastically received by paediatric physiotherapists and parents of infants in their first year. Reviewers highlighted the videos' clear presentation, focused and practical content, achievable objectives and relevance to both clinical practice and home‐based activities. Both physiotherapists and parents reported that the material was easy to implement with readily available items, found the video length suitable and planned to recommend it to others, confirming its usability and appropriateness for the target audience.

## Author Contributions

**Natália Guimarães Melo:** conceptualization, investigation, funding acquisition, writing – original draft, writing – review and editing, visualization, validation, methodology, software, formal analysis, resources, supervision, data curation, project administration. **Nayara Rodrigues Gomes de Oliveira:** conceptualization, investigation, funding acquisition, writing – original draft, writing – review and editing, visualization, validation, methodology, software, formal analysis, project administration, resources, supervision, data curation. **Vanessa Cordeiro de Sousa:** conceptualization, investigation, writing – original draft, methodology, validation, visualization, writing – review and editing, software, formal analysis, supervision, data curation. **Cibelle Kayenne Martins Roberto Formiga:** conceptualization, investigation, funding acquisition, writing – original draft, methodology, validation, visualization, writing – review and editing, software, formal analysis, project administration, resources, supervision, data curation.

## Funding

The author (N.R.G.O.) received a scholarship funded by the Coordenação de Aperfeiçoamento de Pessoal de Nível Superior (CAPES) (SCBA88881.691251/2022‐01).

## Conflicts of Interest

The authors declare no conflicts of interest.

## Supporting information


**Data S1:** Supplementary Information.

**Data S2:** Supplementary Information.

## Data Availability

The raw data supporting the conclusions of this article will be made available by the authors upon request.

## References

[cch70324-bib-0001] Bakilan, F. , G. Sarıçimen , B. Ortanca , et al. 2023. “A Quality Analysis of Cerebral Palsy Videos on Youtube.” Journal of Physical Medicine & Rehabilitation Sciences 26, no. 3: 299–305.

[cch70324-bib-0002] Barbosa, R. F. M. , A. K. L. L. Gonzaga , F. A. Jardim , K. D. S. Mendes , and N. O. Sawada . 2023. “Methodologies Used by Nursing Professionals in the Production of Educational Videos: An Integrative Review.” Revista Latino‐Americana de Enfermagem 31, no. 2: 3950. 10.1590/1518-8345.6690.3951 PMC10243433 37283420

[cch70324-bib-0003] Blee, S. M. , J. Facdol , M. D. Dixon , V. Master , J. M. Switchenko , and R. D. Pentz . 2022. “Dissemination of Validated Health Literacy Videos: A Tailored Approach.” Cancer Medicine 11, no. 7: 1678–1687. 35107221 10.1002/cam4.4572 PMC8986138

[cch70324-bib-0004] Cavalcante, J. C. , J. Cortes Cavalcante , M. Faria Sales , et al. 2024. “Analysis of the Brazilian‐Portuguese Content on Autism Spectrum Disorder Available on YouTube Videos.” Physical & Occupational Therapy in Pediatrics 44, no. 1: 128–142. 37069791 10.1080/01942638.2023.2199843

[cch70324-bib-0005] Corrêa, B. D. S. O. , F. G. B. Góes , A. C. S. S. D. Silva , et al. 2024. “Effectiveness of Educational Technology in Video Format on Home Bathing of Term Newborns.” Texto & Contexto – Enfermagem 33, no. 1: 1–13.

[cch70324-bib-0006] de Campos, D. C. , L. F. da Silva , A. T. Reis , F. G. B. Góes , J. R. M. M. de Moraes , and R. C. B. de Aguiar . 2021. “Development and Validation of an Educational Video to Prevent Falls in Hospitalized Children.” Texto & Contexto – Enfermagem 30: e20190238.

[cch70324-bib-0007] Fernandes, J. S. , A. P. Duca , J. Cemin , et al. 2022. “Survey of Risk Indicators for Child Development in a Primary Health Care Program: A Speech‐Language Pathology Perspective.” Distúrbios da Comunicação 34, no. 3: e53847.

[cch70324-bib-0008] Formiga, C. K. M. R. , M. E. B. Vieira , R. R. Fagundes , and M. B. M. Linhares . 2017. “Predictive Models for Early Motor Development of Preterm Infants: A Prospective Longitudinal Study.” Journal of Human Growth and Development 27, no. 2: 189–197.

[cch70324-bib-0009] Fuentefria, R. N. , R. C. Silveira , and R. S. Procianoy . 2017. “Motor Development of Preterm Infants Assessed by the Alberta Infant Motor Scale: Systematic Review Article.” Jornal de Pediatria 93, no. 4: 328–342. 28506665 10.1016/j.jped.2017.03.003

[cch70324-bib-0010] Furtado, M. A. S. , R. R. Sousa Junior , L. A. Soares , et al. 2022. “Analysis of Informative Content on Cerebral Palsy Presented in Brazilian‐Portuguese YouTube Videos.” Physical & Occupational Therapy in Pediatrics 42, no. 4: 369–383. 35253603 10.1080/01942638.2022.2046677

[cch70324-bib-0011] Hadders‐Algra, M. 2018. “Early Human Motor Development: From Variation to the Ability to Vary and Adapt.” Neuroscience and Biobehavioral Reviews 90, no. 1: 411–427. 29752957 10.1016/j.neubiorev.2018.05.009

[cch70324-bib-0012] Higgs, E. S. , A. B. Goldberg , A. B. Labrique , et al. 2014. “Understanding the Role of mHealth and Other Media Interventions for Behavior Change to Enhance Child Survival and Development in Low‐ and Middle‐Income Countries: An Evidence Review.” Journal of Health Communication 19, no. Suppl. 1: 164–189. 25207452 10.1080/10810730.2014.929763 PMC4255285

[cch70324-bib-0013] Khurana, S. , A. E. Kane , S. E. Brown , T. Tarver , and S. C. Dusing . 2020. “Effect of Neonatal Therapy on the Motor, Cognitive, and Behavioral Development of Infants Born Preterm: A Systematic Review.” Developmental Medicine and Child Neurology 62, no. 6: 684–692. 32077096 10.1111/dmcn.14485 PMC7920849

[cch70324-bib-0014] Lee, H. Y. , A. Q. Zhou , R. M. Lee , and A. L. Dillon . 2020. “Parents' Functional Health Literacy Is Associated With Children's Health Outcomes: Implications for Health Practice, Policy, and Research.” Children and Youth Services Review 110: 104801.

[cch70324-bib-0015] Luoto, J. E. , I. Lopez Garcia , F. E. Aboud , et al. 2021. “Group‐Based Parenting Interventions to Promote Child Development in Rural Kenya: A Multi‐Arm, Cluster‐Randomised Community Effectiveness Trial.” Lancet Global Health 9, no. 3: e309–e319. 33341153 10.1016/S2214-109X(20)30469-1 PMC8054650

[cch70324-bib-0016] Meyers, N. , A. F. Glick , A. L. Mendelsohn , et al. 2020. “Parents' Use of Technologies for Health Management: A Health Literacy Perspective.” Academic Pediatrics 20, no. 1: 23–30. 30862511 10.1016/j.acap.2019.01.008 PMC6733672

[cch70324-bib-0017] Muhoozi, G. K. M. , P. Atukunda , L. M. Diep , et al. 2018. “Nutrition, Hygiene, and Stimulation Education to Improve Growth, Cognitive, Language, and Motor Development Among Infants in Uganda: A Cluster‐Randomized Trial.” Maternal & Child Nutrition 14, no. 2: e12527. 28925580 10.1111/mcn.12527 PMC6866193

[cch70324-bib-0018] Musiimenta, A. , W. Tumuhimbise , N. Pinkwart , J. Katusiime , G. Mugyenyi , and E. C. Atukunda . 2021. “A Mobile Phone‐Based Multimedia Intervention to Support Maternal Health Is Acceptable and Feasible Among Illiterate Pregnant Women in Uganda: Qualitative Findings From a Pilot Randomized Controlled Trial.” DIGITAL HEALTH 7: 2055207620986296. 33717497 10.1177/2055207620986296 PMC7917428

[cch70324-bib-0019] Pedersen, M. R. L. , and A. F. Hansen . 2022. “Interventions by Caregivers to Promote Motor Development in Young Children: Caregivers' Attitudes and Benefits Hereof—A Scoping Review.” International Journal of Environmental Research and Public Health 19, no. 18: 1–14. 10.3390/ijerph191811543 PMC9517187 36141815

[cch70324-bib-0020] Pluye, P. , A. Tskhay , C. Loignon , et al. 2025. “Fathers and Mothers With Low Socioeconomic Status Anticipate More Benefits From Trustworthy Easy‐to‐Read Online Child‐Related Information Compared to Other Parents: The 4‐Year IAM Prospective Time Series.” Maternal and Child Health Journal 29, no. 1: 126–137. 39630399 10.1007/s10995-024-04023-0 PMC11805876

[cch70324-bib-0021] Rosas‐Blum, E. D. , H. M. Granados , B. W. Mills , and M. Leiner . 2018. “Comics as a Medium for Parent Health Education: Improving Understanding of Normal 9‐Month‐Old Developmental Milestones.” Frontiers in Pediatrics 6: 203. 30073157 10.3389/fped.2018.00203 PMC6060568

[cch70324-bib-0022] Santos, A. S. , A. D. S. Santos , L. D. N. Rodrigues , et al. 2020. “Construction and Validation of an Educational Technology for Mother‐Child Bond in the Neonatal Intensive Care Unit.” Revista Brasileira de Enfermagem 73, no. 4: e20190083. 32490997 10.1590/0034-7167-2019-0083

[cch70324-bib-0023] Schneider, L. , M. Kosola , K. Uusimäki , et al. 2021. “Mothers' Perceptions on and Learning From Infant and Young Child‐Feeding Videos Displayed in Mother and Child Health Centers in Kenya: A Qualitative and Quantitative Approach.” Public Health Nutrition 24, no. 12: 3845–3858. 34034846 10.1017/S1368980021002342 PMC10195386

[cch70324-bib-0024] Silva, C. F. R. , A. L. R. Greco , A. M. C. Vieira , L. R. Machado , G. Sgandurra , and E. Tudella . 2026. “Contextual Risk Factors for Atypical Motor Development in Infants Exposed to Poverty: A Longitudinal Study.” Acta Psychologica 262: 106054. 41344174 10.1016/j.actpsy.2025.106054

[cch70324-bib-0025] Trubian, F. , M. Zimmermann , C. C. Sangali , et al. 2022. “Follow‐Up do Desenvolvimento Motor de Prematuros: Impacto das Orientações Parentais.” Revista de Ciências Médicas e Biológicas 21, no. 1: 46–52.

[cch70324-bib-0026] Zhang, D. , L. Jin , D. Liang , et al. 2020. “Assessing Feasibility of an Early Childhood Intervention Using Mobile Phones Among Low‐Income Mothers of Newborns: Qualitative Interview Study.” JMIR Formative Research 4, no. 5: e17179. 32463374 10.2196/17179 PMC7290447

[cch70324-bib-0027] Zubler, J. M. , L. D. Wiggins , M. M. Macias , et al. 2022. “Evidence‐Informed Milestones for Developmental Surveillance Tools.” Pediatrics 149, no. 3: e2021052138. 35132439 10.1542/peds.2021-052138 PMC9680195

