# Supplementary material for: Move and Play Programme: Perceptions of Paediatric Physiotherapists and Parents of Infants
Source: Child Care Health Dev. 2026 Aug 5;52(5):e70324. doi: 10.1111/cch.70324 (PMC13439336; doi:10.1111/cch.70324)
Supplement: Supplementary file 1 [file CCH-52-e70324-s001.docx]

**Supplementary Material 1: Questionnaire for Physiotherapists**

**FIRST STEP: IDENTIFICATION OF THE PHYSIOTHERAPIST**

1) **Full name:**

2) **Which category do you identify with?**

(	) Fisioterapeuta que trabalha com criança

**3) Sex?**

( ) Female ( ) Male ( ) Other

**4) Age: ______ (years)**

**5) Civil Status:**

( ) Single ( ) Married ( ) Divorced ( ) Widowed

**6) Educational Level:**

( ) Doctoral Degree (Completed)

( ) Doctoral Degree (In Progress)

( ) Master’s Degree (Completed)

( ) Master’s Degree (In Progress)

( ) Postgraduate Certificate/Diploma (Completed)

( ) Postgraduate Certificate/Diploma (In Progress)

( ) Bachelor’s Degree (Completed)

( ) Bachelor’s Degree (In Progress)

**8) How long have you been practicing since graduation?**

**9) How long have you been working with children? __________**

**10) Do you work with follow-up programs for high-risk infants?**

( ) Yes ( ) No

**11) Have you ever worked with telehealth or remote monitoring for children?**

( ) Yes ( ) No

**12) State of residence:**

**13) City of residence:**

**SECOND STAGE: EVALUATION OF THE EDUCATIONAL MATERIAL**

In this stage, you will watch the video available below and complete the form with the questions that follow. Please provide your opinion according to what best represents each of the criteria below. There are three types of response formats:

- Yes or No;
- A score from 0 to 10, where 0 is the lowest rating and 10 is the highest;
- Totally adequate, adequate, partially adequate, and inadequate.

There are no right or wrong answers. What matters is your opinion. If possible, please answer all items.

**PRESENTATION**

**1) Is the title appropriate for the video content?**

( ) Totally adequate ( ) Adequate ( ) Partially adequate ( ) Inadequate

**2) Was the audio quality adequate to allow you to hear the instructions?**

( ) Totally adequate ( ) Adequate ( ) Partially adequate ( ) Inadequate

**3) Was the professional’s positioning adequate for viewing the stimulus?**

( ) Totally adequate ( ) Adequate ( ) Partially adequate ( ) Inadequate

**4) Was the baby’s position adequate for viewing the stimulus?**

( ) Totally adequate ( ) Adequate ( ) Partially adequate ( ) Inadequate

**5) Was the video duration adequate to demonstrate the activity?**

( ) 0 ( ) 1 ( ) 2 ( ) 3 ( ) 4 ( ) 5 ( ) 6 ( ) 7 ( ) 8 ( ) 9 ( ) 10

**VIDEO CONTENT**

**6) Was the therapist’s explanation of the objective clear and concise?**

( ) Totally adequate ( ) Adequate ( ) Partially adequate ( ) Inadequate

**7) Were the instructions on how to perform the stimulus clear and concise?**

( ) Totally adequate ( ) Adequate ( ) Partially adequate ( ) Inadequate

**8) Was the video adequate for you to be able to reproduce the activity?**

( ) Totally adequate ( ) Adequate ( ) Partially adequate ( ) Inadequate

**9) Was the language used appropriate?**

( ) Totally adequate ( ) Adequate ( ) Partially adequate ( ) Inadequate

**10) Was the resource (toy or instrument) used in the activity appropriate?**

( ) Totally adequate ( ) Adequate ( ) Partially adequate ( ) Inadequate

**OBJECTIVE**

**11) Is the video appropriate for the proposed objective?**

( ) Totally adequate ( ) Adequate ( ) Partially adequate ( ) Inadequate

**12) Is the video appropriate for teaching this activity or stimulus?**

( ) Totally adequate ( ) Adequate ( ) Partially adequate ( ) Inadequate

**FEASIBILITY IN CLINICAL PRACTICE**

**13) Would you find these videos useful in your clinical practice?**

( ) Yes	( ) No

**14) Would you recommend this material to a patient you are currently following?**

( ) Yes	( ) No

**15) When providing guidance to families for home-based activities, do you think this material would be useful?**

( ) Yes	( ) No

**16) If you were to conduct telemonitoring or telehealth sessions, do you believe you could use this material as a resource?**

( ) Yes	( ) No

**17) Do you believe that the families of your patients would have ease of access to this material?**

( ) Yes	( ) No

**18) Do you believe that the families of your patients would be able to understand the content of this material?**

( ) 0 ( ) 1 ( ) 2 ( ) 3 ( ) 4 ( ) 5 ( ) 6 ( ) 7 ( ) 8 ( ) 9 ( ) 10

**COMMENTS AND SUGGESTIONS**

**19) Please leave your comments, suggestions, or feedback here:**
