# Supplementary material for: Move and Play Programme: Perceptions of Paediatric Physiotherapists and Parents of Infants
Source: Child Care Health Dev. 2026 Aug 5;52(5):e70324. doi: 10.1111/cch.70324 (PMC13439336; doi:10.1111/cch.70324)
Supplement: Supplementary file 2 [file CCH-52-e70324-s002.docx]

**Supplementary Material 2: Questionnaire for the infant’s parents**

**FIRST STAGE: PARTICIPANT IDENTIFICATION**

1) **Full name:**

**2) Which category do you identify with?**

( ) Parent of a child up to 1 year old	( ) Caregiver of a child up to 1 year old

**3) Sex:**

( ) Female ( ) Male ( ) Other

**4) Age: ______ (years)**

**5) Civil Status:**

( ) Single ( ) Married ( ) Divorced ( ) Widowed

**6) Educational Level:**

( ) Postgraduate Diploma/Certificate (Completed)

( ) Postgraduate Diploma/Certificate (In Progress)

( ) Bachelor’s Degree (Completed)

( ) Bachelor’s Degree (In Progress)

( ) Secondary Education (Completed)

( ) Secondary Education (Incomplete)

( ) Primary Education (Completed)

( ) Primary Education (Incomplete)

**7) Occupation:**

**8) How many children do you have? __________**

**9) How many of your children are up to 1 year old? _______**

**10) Do you have children or care for a child who requires follow-up due to complications during pregnancy or childbirth?**

( ) Yes ( ) No

**11) How many children (or children you care for) required follow-up due to complications during pregnancy or childbirth?**

**12) Do you need to perform activities at home with your child (or the child you care for) to support their development?**

( ) Yes ( ) No

**13) Have you had difficulty performing activities at home with your child (or the child you care for) to support their development?**

( ) Yes ( ) No

**14) Does your child (or the child you care for) receive medical follow-up?**

( ) Yes ( ) No

**15) Does your child (or the child you care for) receive follow-up care from a physical therapist, speech therapist, psychologist, or occupational therapist?**

( ) Yes ( ) No

**16) Have these professionals ever provided you with instructional materials or guidance?**

( ) Yes ( ) No

**17) Have you had difficulty understanding this material or following these instructions?**

( ) Yes ( ) No

**18) If yes, what were the reasons?**

( ) I did not have the necessary materials

( ) I did not understand the instructions

( ) The material or instructions used difficult language

( ) I did not have enough time

( ) Other

**19) State of residence: ______**

**20) City of residence: ______**

**SECOND STAGE: EVALUATION OF THE EDUCATIONAL MATERIAL**

In this stage, you will watch the video below and then evaluate it using the questions that follow. Please provide your opinion according to what best represents each of the criteria:

- Totally adequate, Adequate, partially adequate or Inadequate
- 1 – Yes or No
- Score from 0 to 10: When a question uses this scale, consider 0 as the lowest rating for that criterion and 10 as the highest.

There are no right or wrong answers. What matters is your opinion. If possible, please answer all items.

**PRESENTATION**

**1) Is the title appropriate for the video content?**

( ) Totally adequate ( ) Adequate ( ) Partially adequate ( ) Inadequate

**2) Was the audio clear enough for you to hear the instructions?**

( ) Totally adequate ( ) Adequate ( ) Partially adequate ( ) Inadequate

**3) Was the professional’s position appropriate for you to see the activity clearly?**

( ) Totally adequate ( ) Adequate ( ) Partially adequate ( ) Inadequate

**4) Was the baby’s position appropriate for you to see the activity clearly?**

( ) Totally adequate ( ) Adequate ( ) Partially adequate ( ) Inadequate

**5)** **Was the video length appropriate to show the activity?**

( ) 0 ( ) 1 ( ) 2 ( ) 3 ( ) 4 ( ) 5 ( ) 6 ( ) 7 ( ) 8 ( ) 9 ( ) 10

**VIDEO CONTENT**

**6) Was the therapist’s explanation of the objective clear and easy to understand?**

( ) Totally adequate ( ) Adequate ( ) Partially adequate ( ) Inadequate

**7) Were the instructions on how to perform the activity clear and easy to understand?**

( ) Totally adequate ( ) Adequate ( ) Partially adequate ( ) Inadequat

**8) Was the video adequate for you to carry out the activity at home?**

( ) Totally adequate ( ) Adequate ( ) Partially adequate ( ) Inadequat

**9) Was the language used appropriate?**

( ) Totally adequate ( ) Adequate ( ) Partially adequate ( ) Inadequat

**10) Was the toy or material used in the activity appropriate?**

( ) Totally adequate ( ) Adequate ( ) Partially adequate ( ) Inadequat

**OBJECTIVE**

**11) Is the video appropriate for demonstrating the proposed activity?**

( ) Totally adequate ( ) Adequate ( ) Partially adequate ( ) Inadequat

**12)** **Is the video appropriate for teaching this activity or stimulus?**

( ) Totally adequate ( ) Adequate ( ) Partially adequate ( ) Inadequat

**HOME APPLICABILITY**

**13) Do you believe these videos will be useful to support your child’s development?**

( ) Yes ( ) No

**14) Would you recommend these videos to a friend or family member with a child?**

( ) Yes ( ) No

**15) Were you able to perform the activity from the video with your child?**

( ) Yes ( ) No

**16) Did you find it difficult to obtain the materials or toys used in the videos?**

( ) Yes ( ) No

**17) Do you think it is possible to perform the same activity using materials or toys you already have at home?**

( ) Yes ( ) No

**18) Do you think you would have time to perform this activity at home with your child?**

( ) Yes ( ) No

**19) Were the instructions sufficient for you to carry out the activity with your child?**

( ) 0 ( ) 1 ( ) 2 ( ) 3 ( ) 4 ( ) 5 ( ) 6 ( ) 7 ( ) 8 ( ) 9 ( ) 10

**20) Did you need to watch the video more than once to understand the instructions?**

( ) Yes ( ) No

**COMMENTS AND SUGGESTIONS**

**21) Please leave your comments or feedback here:**
